# A Rapid and Sensitive Detection of HIV-1 with a One-Pot Two-Stage Reverse Transcription Recombinase Aided Real-Time PCR Assay

**DOI:** 10.3390/tropicalmed8020105

**Published:** 2023-02-06

**Authors:** Fengyu Tian, Cong Jin, Shangzhi Ji, Yanqing Tie, Guohao Fan, Ruiqing Zhang, Yehuan Zheng, Xinxin Shen, Xuejun Ma, Zhishan Feng

**Affiliations:** 1 Graduate School, Hebei Medical University, Shijiazhuang 050031, China; 2 Department of Clinical Laboratory, Hebei General Hospital, Shijiazhuang 050070, China; 3 NHC Key Laboratory of Medical Virology and Viral Diseases, National Institute for Viral Disease Control and Prevention, Chinese Center for Disease Control and Prevention, Beijing 102206, China; 4 National AIDS Reference Laboratory, National Center for AIDS/STD Control and Prevention, Chinese Center for Disease Control and Prevention, Beijing 102206, China; 5 Beijing Wantai Biological Pharmacy Enterprise Co., Ltd., Beijing 102206, China; 6 Hebei Key Laboratory of Molecular Medicine, Shijiazhuang 050070, China; 7 Hebei Clinical Research Center for Laboratory Medicine, Shijiazhuang 050070, China; 8 Autobio Diagnostics Co., Ltd., Zhengzhou 450000, China

**Keywords:** HIV-1, RT-RAA, qRT-PCR, clinical detection

## Abstract

Human immunodeficiency virus 1 (HIV-1) attacks the immune system, making people susceptible to various diseases, thus increasing their risk of death. Comprehensive detection of major HIV-1 strains circulating in China is vital for effective HIV-1 infection prevention and treatment. HIV-1 nucleic acid detection is considered effective for HIV-1 diagnosis since traditional immunological testing may fail to detect HIV-1 infection during the window period. This work demonstrates a one-pot two-stage amplification assay (RT-RAP), a combination of reverse transcription recombinase (RT- RAA), and quantitative real-time polymerase chain reaction (qRT-PCR). The turn-around time of the assay is only 50 min and can be performed with commonly available laboratory equipment, the qPCR devices. The RT-RAP assay could detect approximately 5 and 14 copies/reaction of HIV-1 DNA and RNA using recombinant plasmids and standard reference strains, respectively. Additionally, we found that the clinical performance of RT-RAP (detected 169 samples out of 170 specimens) was consistent with that of qRT-PCR. The sensitivity and specificity of RT-RAP were 100.00% (99/99) and 98.59% (70/71), respectively, while its positive and negative predictive values were 99.00% (99/100) and 100.00% (70/70), respectively. The total coincidence rate of the RT-RAP was 99.41% (169/170), with a kappa value of 0.988 (*p* < 0.05). We demonstrated that RT-RAP could rapidly detect the common HIV-1 subtypes commonly circulating in China with comparable sensitivity and specificity to qRT-PCR.

## 1. Introduction

Human immunodeficiency virus 1(HIV-1), the etiological agent of acquired immunodeficiency syndrome (AIDS), belongs to the genus Lentivirus of the retroviridae family [1]. HIV-1 is mainly characterized by its infection latency and subtypes diversity [2]. Most HIV/AIDS cases are associated with the subtypes of the major group, including A, B, C, D, F, G, H, and J, in addition to the unique recombinant forms (URFs) and over 100 circulating recombinant forms (CRFs) globally [3,4]. The increasing HIV-1 diversity across geographic populations poses significant health challenges to many countries, including China. It is estimated that 94 HIV-1 strains have emerged in China over the past two decades, with the predominant strains shifting from CRE_07BC and Subtype B to CRE_01AE and CRE_07BC from CRE_07BC and Subtype B [2]. Detection of these major strains is vital for effective HIV prevention and treatment. Notably, early detection is critical for high-risk groups, infected infants, and monitoring of antiretroviral therapy.

Currently, HIV-1 diagnosis is mainly achieved through a fourth-generation assay (HIV antigen/antibody combination assay) and western blot as a confirmatory assay [5]. Detection of the HIV p24 antigen is very effective for early HIV diagnosis because the antigen appears in the blood during the early stages of HIV infection [6]. Although the assay indeed shortens the window period, it has reportedly failed to diagnose a few acute infection periods characterized by the undetectable p24 antigen but the present HIV nucleic acids [7]. Therefore, such traditional immunological tests will likely fail to detect the virus when the antibody and antigen detection sensitivity is low, highlighting the diagnostic value of the HIV nucleic acid detection [8].

Currently, quantitative real-time PCR (qRT-PCR) is regarded as the gold standard for HIV-1 detection. Many qRT-PCR methods with excellent sensitivity for HIV-1 are commercially available [9,10,11,12,13,14,15]. However, these qRT-PCR assays have a relatively high cost, with longer turn-around time. These limitations have partly prompted the development of isothermal nucleic acid amplification, which is relatively fast and does not rely upon complex thermal cycling parameters. Many isothermal nucleic acid amplification assays for HIV-1 detection have been reported [16,17,18,19,20,21]. For example, recombinase-aided amplification (RAA) with a detection limit of 20 copies per reaction was used for HIV-1 detection in a 30-min reaction [21]. However, this study only detected a few circulating HIV subtypes in China, partly due to the difficulty in designing a longer (46–52 mer) and conservative RAA probe targeting diverse HIV subtypes.

We previously developed a novel recombinase-aided PCR (RAP) method, enabling real-time amplification and ultrasensitive rapid detection of respiratory pathogens [22]. The principle of this RAP method was to first pre-amplify a small amount of the target gene fragments with RAA and then amplify the RAA products using qPCR for real-time and fast detection. However, the RAP method had relatively higher operational requirements since RAA occurred on the inner surface of the tube cap, necessitating maintenance of the circular liquid drop on the surface to ensure the efficiency of RAA. Additionally, the pre-added magnesium could also affect RAA efficiency. To overcome these RAP shortcomings, in the present study, we optimized the reverse transcription RAP (RT-RAP) using a single-tube temperature-adjustable block to achieve one-pot and two-stage amplification for the detection of HIV-1.

## 2. Materials and Methods

### 2.1. Sample Collection and Nucleic Acid Extraction

A total of 100 stock serum samples from HIV-1-infected patients and 70 healthy HIV-1-seronegative blood bank donors were preserved in the National Center for AIDS/STD Control and Prevention and the National Institute for Viral Disease Control and Prevention of the Chinese Center for Disease Control and Prevention. HIV RNA was extracted from the samples using a DNA/RNA extraction kit (Lizhu, Zhuhai, China) according to the manufacturer’s instructions. The stock nucleic acids of blood-borne or sex-transmitted pathogens, including hepatitis B virus (HBV), hepatitis C virus (HCV), Chlamydia trachomatis (CT), and Treponema pallidum (TP), were provided by the National Institute for Viral Disease Control and Prevention of the Chinese Center for Disease Control and Prevention. The purified RNAs were stored at −80° until used.

### 2.2. Primer and Probe Design of RT-RAP Assay

A total of 389 whole genome sequences of six HIV-1 subtypes common in China (170 CRF_01AE, 38 CRF_07BC, 57 Subtype B, 9 Subtype C, 56 Subtype BC, and 35 CRF_08BC) and 24 other CRFs rare in China were downloaded from the Los Alamos National Laboratory HIV database. These sequences were aligned and analyzed for high homology conserved sequences using CLUSTAL W (BioEdit software 7.0.9.0, Manchester, Greater Manchester, England). The conserved oligonucleotides in the Gag and Pol regions were selected as the candidate sequences for designing the RAA forward and reverse primers, which were then evaluated using Oligo7 software. The PCR forward and reverse primers and probes derived from previously published assays [9,10] served as internal primers for RAP. The primers and probes were synthesized and purified by Shanghai Bioengineering (Shanghai, China), and their details are listed in Table 1.

### 2.3. Plasmid Construction

According to their geographical distribution in the Los Alamos database, the high-to-low proportion order of the six HIV-1 subtypes (common in China) was CRF_01AE, CRF_07BC, subtype B, subtype C, subtype BC, and CRF_08BC. Since subtype B and C sequences had similar primers and probe locations, they shared a common set of plasmids. A 174 bp Gag region fragment (referred to as HXB2 reference strain:1253–1426) and a 219 bp Pol region fragment (referred to as HXB2 reference strain:4885–5103) of the six subtypes were cloned, respectively, into plasmid vector pUC57. Researchers have found no significant difference in PCR efficiency between linearized and cyclic plasmid DNA. The circular plasmids are less susceptible to degradation in the long-term [23]. Actually, the plasmid DNA sample is a mixture of supercoiled, nick circular, and linearized forms. Following up with the same strategy of Chih-Hui Lin’s and others’ papers [24,25,26,27,28,29], in our present study, we directly quantified (averaged by five measurements) the plasmid DNA sample without enzyme treatment to make a rough and reasonable estimation. We think this may reduce concentration estimation error to some extent by averaging different conformation forms and does not significantly impact the conclusions of our study. So, these recombinant plasmids were then directly quantified with Qubit™dsDNA HS Assay Kit (Thermo Fisher Scientific, Waltham, MA, United States) and converted to copy numbers using the following formula: DNA copy number (copy number/μL) = {[6.02 × 10^23^ × plasmid concentration (ng/μL) × 10^−9^]}/[DNA length × 660]. Thereafter, the plasmids were diluted with TE buffer from 10^0^–10^9^ copies/2μL and stored at −30 °C until use.

### 2.4. Solid Docosane Barrier

Docosane (Aladdin, Shanghai, China) exists as an insoluble solid at room temperature. However, when the temperature is above 44 °C, it melts, forming a molten docosane wax that can be used for sealing the qPCR mix. Through multiple comparisons, we explored the optimal amount of docosane (ranging from 10 μL to 45 μL) for separating the first-stage and second-stage reactions of the RAP assay.

### 2.5. One-Pot Two-Stage RT-RAP Assay

**Figure 1:**
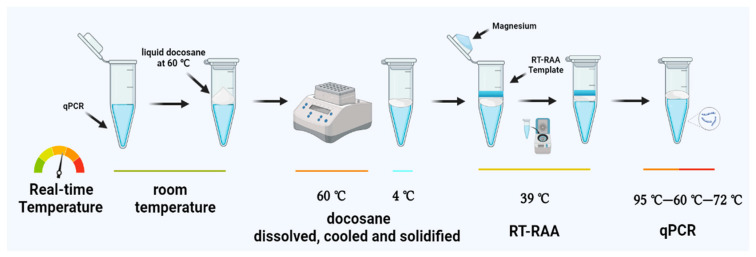
An overview of the one-pot two-stage RT-RAP. A 40 μL qPCR mix was added in a qPCR tube, followed by 25 μL of liquid docosane for wax sealing upon cooling. Magnesium, template, and RT-RAA mix were short-centrifuged to activate RT-RAA assay, and the RT-RAA products were used as templates for the subsequent qPCR.

The RT-RAP assay was performed in two stages within a single tube. The tube was divided into two parts using a heat-removable docosane barrier. Briefly, the docosane was molten at 60 °C, and 25 μL of liquid docosane was added into the qPCR tube containing 40 μL of the qPCR mix. The docosane was then solidified to form a block sealing on the sample. Thereafter, 8 μL of RT-RAA mix and 2 μL of the templates were added to the block surface, and 1 μL 140 mM magnesium acetate was suspended on the inner surface of the tube lid before covering the tubes. The mixture was short-centrifuged to initiate the RT-RAA, and the RT-RAA products were then used as templates for the subsequent qPCR. The RT-RAA (total 8 μL) included 400 nM of each RAA primer and RAA reaction buffer (Jiangsu Qitian, Wuxi, China). The qPCR (total 40 μL) contained 500 nM of each PCR primer and 250 nM of the probe, 150 μM of dNTPs, and 4 × qPCR buffers (Entrans qPCR Probe Set V2, ABclonal, Wuhan, China). The RT-RAP reaction conditions were as follows: 39 °C for 15 min, 95 °C for 5 min, followed by 20 cycles of 95 °C for 15 s, 60 °C for 30 s, and 72 °C for 30 s. The scheme of the one-pot two-stage RT-RAP is shown in Figure 1.

As part of the optimization process, we tested different volumes of docosane, time for RT-RAA incubation, and primer-probe ratio using 10 copies of plasmid as a template. We also evaluated the qPCR annealing temperature using 10^4^ copies of plasmid as a template, aiming to make the fluorescence curves more typical and the results easier to distinguish based on previous studies in our laboratory.

### 2.6. Sensitivity, Specificity, and Reproducibility of the RT-RAP Assay

To test the sensitivity of RT-RAP in detecting DNA, we used five recombinant plasmids (corresponding to the six common subtypes of HIV-1) with a serial dilution range of 10^0^ to 10^5^ copies/2 μL DNA as the templates of RT-RAP. For RNA detection sensitivity, the RNA extracted from the serial dilutions of HIV international standard strain (Original QC from Rush University, Chicago, Illinois, USA, used for inter-compartmental quality control testing, 150,000 copies/mL to 10–100 copies/2 μL), using the QIAamp Viral RNA Mini Kit (Qiagen, Hilden, Germany) as RNA standard. Each test was repeated eight times to determine the reproducibility of the RT-RAP assays. Additionally, several blood-borne or sex-transmitted pathogens, including HBV, HCV, CT, and TP, were used to evaluate the specificity of the RT-RAP assays.

### 2.7. RT-RAP Detection of Clinical Samples

A total of 100 (88 known and 12 unknown subtypes) HIV-1 positive and 70 negative samples were selected for evaluating the clinical performance of RT-RAP assays for various samples. The detailed viral load of all HIV-1-positive clinical samples was previously measured by Roche Cobas TaqMan HIV-1 v2.0 or Abbott RealTime HIV-1 (m2000sp). These positive samples were also tested in parallel by a commercial HIV-1 qRT-PCR kit (Beijing Wantai, Beijing, China).

### 2.8. Statistical Analysis

All statistical analyses were performed with IBM SPSS Statistics 26 (IBM Corporation, Armonk, NY, USA). We determined the 95% probability detection limit of the RT-RAP assay using probit analysis, and Kappa values (κ) were used to measure the agreement between RT-RAP and qRT-PCR results.

## 3. Results

### 3.1. Optimization of the One-Pot Two-Stage RT-RAP Assays

**Figure 2:**
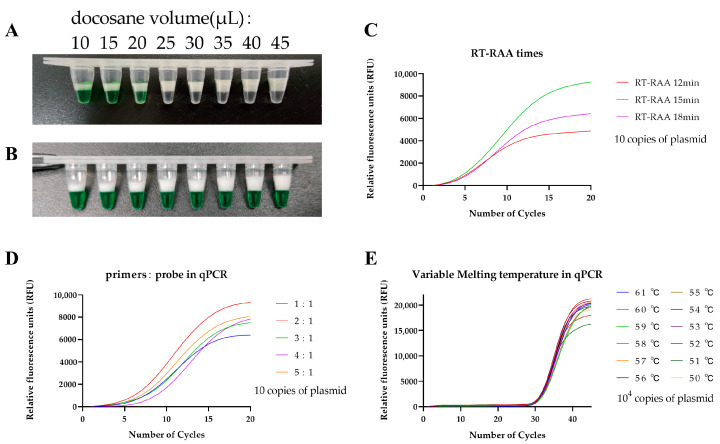
Optimized RT-RAP parameters: (**A**) The optimal volume of docosane simulating RT-RAA invasion (from left to right: 10 μL to 45 μL); (**B**) Thoroughly mixed RT-RAA and qPCR solution at the optimal qPCR annealing temperature (at 60 °C, the reaction was replicated 8 times); (**C**) Relative fluorescence curves of the RT-RAP with different RT-RAA incubation durations (12 min, 15 min, and 18 min); (**D**) Relative fluorescence curve of the RT-RAP with the different volume ratios of the qPCR primers: probes (from 1:1 to 5:1); (**E**) Relative fluorescence curve of the RT-RAP with different annealing temperatures from 50 °C–61 °C. The final optimal conditions for further experimentation were as follows: RT-RAA incubation at 39 °C for 15 min, qPCR annealing at 60 °C, and a primer: probe ratio of 2:1 (500 nM:250 nM).

To determine the docosane block effect under low qPCR temperatures, we used 10 μL of Green GoTaq Flexi Buffer to simulate RT-RAA and added 10 μL to 45 μL of docosane into 40 μL of the qPCR mix. After the mixture had cooled and solidified, we found that the optimal amount of docosane for blocking the RT-RAA invasion at room temperature was 25 μL (Figure 2A). We also observed that Green GoTaq Flexi Buffer was thoroughly mixed with the qPCR mix at 60 °C in all eight replicated experiments after docosane was completely dissolved (Figure 2B). The multiple physical states of docosane (liquid–solid–liquid–solid), controlled via different temperatures to ensure the blocking effect, simplifies the manual operations of the RAP reaction, making the technique more user-friendly than the original RAP. Consequently, we successfully developed a one-pot two-stage RT-RAP with a temperature-adjustable block. The first step of RT-RAP was the RT-RAA reaction, which occurred at temperatures between 39 °C and 42 °C when a solid barrier (docosane) blocked the RAA mix from the qPCR mix. The second step of RT-RAP was the qPCR stage which occurred when the docosane was molten at the PCR denaturing temperature (95 °C), allowing the RT-RAA products to mix with qPCR and amplify in real time.

To determine the optimum duration for the RT-RAA reaction, we adjusted the time for the RT-RAA assay from 12 to 18 min using 10 copies of plasmid as a template (Figure 2C). The peak height of the fluorescence curve increased while the CT values of the qPCR decreased with increasing RT-RAA reaction time. Nevertheless, the efficient qPCR amplification efficiency was achieved when the RT-RAA time was set to 15 min, implying that qPCR amplification efficiency decreased with increasing time due to RT-RAA byproducts build-up. We also found that the optimum volume ratio of the primers: probe in qPCR was 2:1, corresponding to 500 nM:250 nM (Figure 2D). Moreover, we examined the RT-RAP performance at different qPCR annealing temperatures (between 61 °C and 50 °C) using 10^4^ copies of plasmid as a template (Figure 2E). The results showed that 60 °C was the optimal annealing temperature for detecting HIV-1 plasmids.

### 3.2. Sensitivity, Specificity, and Reproducibility of the RT-RAP Assay

**Figure 3:**
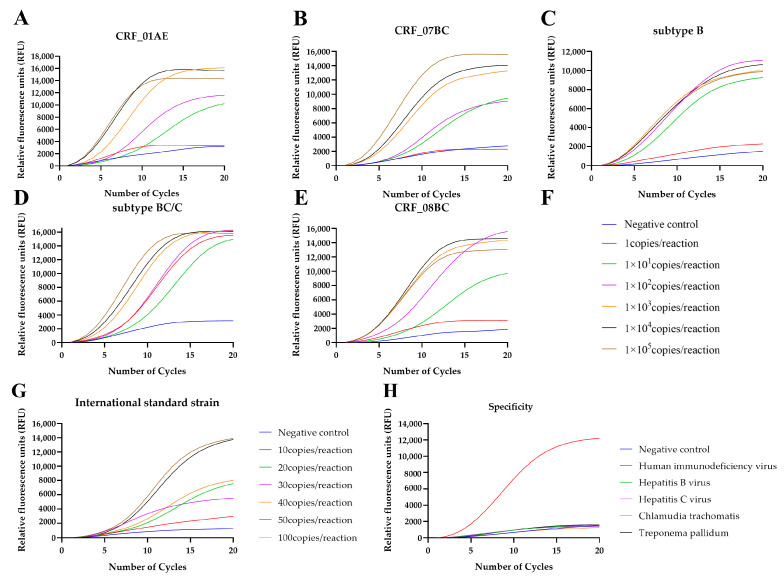
RT-RAP sensitivity evaluation by copy number determination of the plasmids and the international standard strain. The curves with different colors correspond to the amplification efficiency of the qPCR stage of RT-RAP at different template concentrations (plasmids or international standard strain). (**A**–**G**) Sensitivity analysis of the HIV-1 RT-RAP assay. Relative fluorescence curve of HIV-1 CRF01_AE (**A**), CRF07_BC (**B**), Subtype B (**C**), Subtype BC/C (**D**), and CRF08_BC (**E**) at different concentrations (1 to 10^5^ copies/reaction) using RT-RAP. (**F**) Diagram of A–E. (**G**) Relative fluorescence curve of HIV international standard strain from 10 to 100 copies/reaction using RT-RAP. (**H**) Specificity analysis of HIV-1 RT-RAP assay for the common blood-borne and sex-transmitted pathogens.

RT-RAP detected 10 copies/reactions of HIV-1 plasmids and the HIV international standard strain (Figure 3A–G; Table 2 and Table 3). According to probit regression analysis, the 95% detection limit of RT-RAP assay for HIV-1 DNA and RNA reached approximately 5 and 14 copies per reaction (*p* < 0.05), respectively (Table 2 and Table 3). The RT-RAP assay for HIV-1 had 100% specificity (Figure 3H) since no cross-reaction was observed among the four common blood-borne and sex-transmitted pathogens.

### 3.3. Viral Loads of Clinical Samples

**Figure 4:**
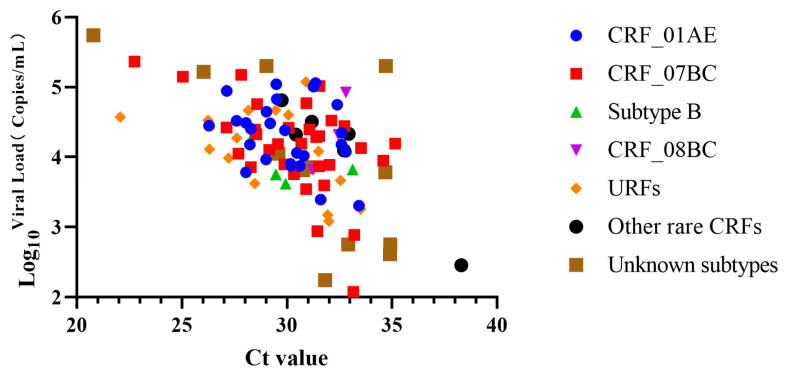
The Ct values and viral load of the clinical samples of HIV-1 CRF_01AE, CRF_07BC, subtype B, CRF_08BC, URFs, and other subtypes. The log10 of viral load was used as the Y-axis, and the positive clinical samples were used as the X-axis.

In this experiment, 100 positive clinical samples were processed, including 27 for CRF_01AE (viral loads of one sample was 2.00 × 10^3^ copies/mL by Abbott kits, but missed by commercial HIV-1 qRT-PCR kit by Beijing Wantai, Beijing, China), 32 for CRF_07BC, 4 for subtype B, 3 for CRF_08BC, 6 for Other rare CRFs, 16 for URFs (unique recombinant forms), and 12 for unknown subtypes. The viral loads determined by the Roche or Abbott kits ranged from 1.19 × 10^2^ copies/mL to 5.49 × 10^5^ copies/mL among the 100 HIV-positive samples. Furthermore, positive samples tested by the commercial kits showed a Ct value ranging from 20.79 to 38.29 (Figure 4).

### 3.4. Clinical Evaluation and Comparison between the RT-RAP and a Commercial qRT-PCR Kit

The RT-RAP and qRT-PCR tests were performed in parallel on 170 samples. The RT-RAP results of 169 samples (99 positives and 70 negatives) were consistent with those of qRT-PCR. One sample was positive by RT-RAP but missed by qRT-PCR, probably due to the ultra-sensitivity of RT-RAP. The RT-RAP and qRT-PCR comparison analysis showed that RT-RAP had 100.00% (99/99) sensitivity and 98.59% (70/71) specificity. We obtained a positive predictive value of 99.00% (99/100), a negative predictive value of 100.00% (70/70), an overall coincidence rate of 99.41% (169/170), and a kappa value of 0.988 (*p* < 0.05) (Table 4).

## 4. Discussion

Per our initial work, conventional RT-qPCR using FAST-Taq polymerase or rapid two-step (denaturation-annealing/extension) working conditions, though fast, did not work well in the presence of a lower virus load. Here, we have developed a one-pot two-stage RT-RAP assay for HIV-1 by combining the rapidity of RT-RAA (an isothermal amplification) with the simplicity of the qPCR probe (traditional “Taqman” three-step qPCR, denaturation–annealing–extension) to ensure the high sensitivity and time-saving. According to the probit regression analysis, the RT-RAP detection sensitivity of the HIV-1 recombinant plasmids and the international standards was approximately 5 and 14 copies per reaction (*p* < 0.05), respectively. The detection limit of 5 copies per reaction was fallen in the range of 1–10 copies, which we think is a rough and reasonable estimation. In the case of using the HIV international standard strain, the diluted concentrations included 10, 20, 30, 40, 50, and 100 copies per reaction (Table 3); the detection limit of 13 copies per reaction was calculated, which we think is a reasonable estimation. The performance evaluation demonstrated that the RT-RAP was comparable with qRT-PCR (100.00% sensitivity, 98.59% specificity, and kappa value of 0.988) in detecting clinical samples with a minimum viral load of 119 copies/mL and a maximum Ct value of 38.29. Furthermore, no cross-reactivity was observed with other blood-borne and sex-transmitted pathogens.

Many qRT-PCR have been developed and successfully applied for HIV-1-specific detection. A few common commercial kits are highly sensitive and could quantify the viral load [11,12,13]. Due to their efficient nucleic acid extraction, these kits reached a sensitivity of 20–60 copies/mL with a loading template volume of up to 20 μL (representing 40% of the total qRT-PCR volume). Although nested PCR uses two pairs of primers to amplify the target gene in two rounds for improved sensitivity, the technique is time-consuming, with a high risk of contamination [30,31]. Loop-mediated isothermal amplification (LAMP), a widely used for isothermal nucleic acid amplification, detected HIV-1 with a sensitivity of 10–100 copies/reaction [16,17]; however, it required a complex primer design. Furthermore, RAA also demonstrated good sensitivity, rapidity, and portability in HIV-1 detection, but its clinical evaluation was inadequate because the subtypes of the HIV-1 clinical samples tested were not clearly described [21].

It has been previously demonstrated that targeting two genomic regions is more suitable for detecting HIV-1 strains than targeting single genes. This is because the mutations in one target can be compensated by mutations in the other, thus reducing the chances of getting false negative results [32]. As most qRT-PCR assays, the proposed RT-RAP assay adopted the same two-target strategy; however, the latter allows for accurate detection within less than one hour, which is faster than normal qRT-PCR. Moreover, RT-RAP bypasses the stringent requirements of conservative and lengthy probes with real-time RAA. Most laboratories can use RT-RAP due to its compatibility with most real-time fluorescent qPCR devices and the independence of the isothermal detection equipment. Previous studies have reported a molecular assay called Penn-RAMP, conducted in a single pot with two compartments separated by docosane (a temperature-controlled barrier) [33]. To optimize our previously reported RAP, we also used docosane as a barrier, ensuring the effectiveness of RT-RAA amplification efficiency during the initial stage of the reaction. This also reduced the experimental operation requirement and the risk of nucleic acid contamination compared with the original “tube with cap” RAP. Additionally, we evaluated the pre-made RT-RAP system and found that the sensitivity of the RT-RAP assay could be maintained for up to two weeks after cryogenic storage by configuring the qPCR system in advance. This provides a supportive basis for the future large-scale application of RT-RAP. Unlike the HIV-positive clinical samples tested by other studies, the clinical samples used in this study had detailed information on the virus subtypes and viral loads (Figure 4). Therefore, our results demonstrated the utility potential of RT-RAP in HIV-1 infection screening using the common subtypes circulating in China.

Despite these interesting findings, there were some limitations to this study. The RT-RAP assay could not accurately quantify HIV-1 viral copy numbers because of the rapidity and qualitative nature of the RT-RAA stage [22]. Furthermore, the current RT-RAP assay only supports 2 μL of the templates, which is one-tenth the volume of qRT-PCR though the detection sensitivity of RT-RAP is equivalent to qRT-PCR in this case. There was no exogenous internal control to monitor the whole reaction in the present study, and this may also serve as a direction for subsequent optimization.

Additionally, more clinical samples are needed to further evaluate the feasibility of RT-RAP assay for more diverse HIV-1 subtypes across China. To enhance the convenience and efficiency of this detection method, integrating the microfluidic chip into RT-RAP may also be a viable option. This would facilitate rapid, sensitive, and high throughput HIV-specific detection at the point-of-care settings.

## 5. Conclusions

In summary, we developed a one-pot and two-stage RT-RAP assay for detecting different HIV-1 strains prevalent in China. The assay can be completed within 50 min using conventional qPCR devices available in most molecular laboratories. Additionally, since this RT-RAP assay combines RAA’s rapidity with the qPCR probe’s simplicity, it exhibits significant sensitivity and specificity, which is crucial for ensuring HIV prevention and treatment efficacy in China.

## Figures and Tables

**Table 1:** Primers and probes sequences used in this study.

Region	Primer/Probe	Sequence (5′—3′)	Source	Position ^a^
Gag	HIV-RAA-F	GAAGTAATACCCATGTTTTCAGCATTATCA	This paper	1288–1317
	HIV-RAA-R	TGCAGCTTCCTCATTGATGGTTTCTTTTAAC	This paper	1419–1389
	HIV-qPCR-F	CATGTTTTCAGCATTATCAGAAGG	[9]	1299–1322
	HIV-qPCR-R	GCTTCCTCATTGATGGTCTCTTT	[9]	1415–1393
	HIV-qPCR-P	FAM-CACCCCACAAGATTTAAACACCATGCTAA-BHQ1	[9]	1326–1354
Pol	HIV-RAA-F	ATTTTCGGGTTTATTACAGGGACAGCAGAGA	This paper	4894–4924
	HIV-RAA-R	CACAATCATCACCTGCCATCTGTTTTCCAT	This paper	5070–5041
	HIV-qPCR-F	GGTTTATTACAGGGACAGCAGAGA	[10]	4901–4924
	HIV-qPCR-R	ACCTGCCATCTGTTTTCCATA	[10]	5060–5040
	HIV-qPCR-P	FAM-ACTACTGCCCCTTCACCTTTCCAGAG-BHQ1	[10]	4978–4953

^a^ Primer/probe positions refer to positions in the genome of the HIV-1 HXB2 reference strain.

**Table 2:** The low detection limit of the RT-RAP assay for HIV-1 DNA.

Copies/Reaction	No. of Positive Samples Tested by the RT-RAP Assays for HIV-1				
	CRF_01AE	CRF_07BC	Subtype B	Subtype C/BC	CRF_08BC
1	1/8	1/8	1/8	2/8	1/8
10^1^	8/8	8/8	8/8	8/8	8/8
10^2^	8/8	8/8	8/8	8/8	8/8
10^3^	8/8	8/8	8/8	8/8	8/8
10^4^	8/8	8/8	8/8	8/8	8/8
10^5^	8/8	8/8	8/8	8/8	8/8
Detection limit (Probit analysis)	5.037	5.037	5.037	4.149	5.037

**Table 3:** The low detection limit of the RT-RAP assay for HIV-1 RNA.

Copies/Reaction	No. of Positive Samples	Ct Values of the Commercial qRT-PCR Kits
10	7/8	36.530
20	8/8	35.615
30	8/8	33.650
40	8/8	34.744
50	8/8	34.022
100	8/8	33.794
Detection limit (Probit analysis)	13.678	

**Table 4:** Sample evaluation comparison between RT-RAP and the commercial qRT-PCR kits.

RT-RAP	Commercial qRT-PCR Kits		Total	Sensitivity (%)	Specificity (%)	PPV (%)	NPV (%)	Kappa
	+	−						
+	99	1	100					
−	0	70	70	100.00	98.59	99.00	100	0.988
Total	99	71	170					

PPV—positive predictive value; NPV—negative predictive value.

## Data Availability

The data sets used and/or analyzed in the current study could be provided on reasonable request. All requests should be made to the correspondence author.
